# Predikin and PredikinDB: a computational framework for the prediction of protein kinase peptide specificity and an associated database of phosphorylation sites

**DOI:** 10.1186/1471-2105-9-245

**Published:** 2008-05-26

**Authors:** Neil FW Saunders, Ross I Brinkworth, Thomas Huber, Bruce E Kemp, Bostjan Kobe

**Affiliations:** 1 School of Molecular and Microbial Sciences, University of Queensland, Brisbane 4072, Australia; 2 St. Vincent's Institute, 41 Victoria Pd, Fitzroy, 3065 Victoria, Australia; 3 Institute for Molecular Bioscience and Special Research Centre for Functional and Applied Genomics, University of Queensland, Brisbane 4072, Australia

## Abstract

**Background:**

We have previously described an approach to predicting the substrate specificity of serine-threonine protein kinases. The method, named Predikin, identifies key conserved *substrate-determining residues *in the kinase catalytic domain that contact the substrate in the region of the phosphorylation site and so determine the sequence surrounding the phosphorylation site. Predikin was implemented originally as a web application written in Javascript.

**Results:**

Here, we describe a new version of Predikin, completely revised and rewritten as a modular framework that provides multiple enhancements compared with the original. Predikin now consists of two components: (i) PredikinDB, a database of phosphorylation sites that links substrates to kinase sequences and (ii) a Perl module, which provides methods to classify protein kinases, reliably identify substrate-determining residues, generate scoring matrices and score putative phosphorylation sites in query sequences. The performance of Predikin as measured using receiver operator characteristic (ROC) graph analysis equals or surpasses that of existing comparable methods. The Predikin website has been redesigned to incorporate the new features.

**Conclusion:**

New features in Predikin include the use of SQL queries to PredikinDB to generate predictions, scoring of predictions, more reliable identification of substrate-determining residues and putative phosphorylation sites, extended options to handle protein kinase and substrate data and an improved web interface. The new features significantly enhance the ability of Predikin to analyse protein kinases and their substrates. Predikin is available at .

## Background

The post-translational modification of proteins by phosphorylation of serine, threonine or tyrosine residues is a ubiquitous process in cellular regulation. Protein kinases, the enzymes responsible for protein phosphorylation, make up almost 2% of protein-encoding genes in the human genome [1] and an estimated 30–50% of human proteins are phosphorylated [2]. Protein kinases and their substrates regulate essentially all cellular processes through complex regulatory networks, in which phosphorylated proteins act as switches that tune the response of the cell to environmental stimuli. Defects in these networks result in a variety of disease states making protein kinases important targets for drug design [3].

In general, a protein kinase acts on a discrete set of substrates to ensure that signalling fidelity is maintained. How a particular protein kinase recognises its substrate protein(s) is therefore a key question. Two major factors determine the formation of a protein kinase-substrate complex [4]. The first, termed substrate recruitment, encompasses any process that increases the effective concentration of the protein kinase substrate. This can be brought about by mechanisms including colocalisation of protein kinase and substrate to a subcellular compartment [5] or complex formation mediated through binding sites either on the protein kinase [6] or a scaffolding protein [7]. The second factor, termed peptide specificity, describes the interaction between amino acid residues in the catalytic domain of the protein kinase and the substrate residues that surround the phosphorylated residue. Crystal structures of protein kinases with bound substrate peptides show that substrate residues at positions -3 to +3 relative to the phosphorylated serine, threonine or tyrosine residue adopt an extended conformation and bind to a pocket in the catalytic domain of the protein kinase [8]. The heptapeptide sequence from -3 to +3 that best binds to the pocket is determined by the physicochemical nature of the residues in the catalytic domain that line the pocket and contact the substrate.

The relative contribution of substrate recruitment and peptide specificity to protein kinase substrate specificity varies between protein kinases. However, it is recognised that for many protein kinase families, particularly those that phosphorylate Ser/Thr residues, peptide specificity is the major factor that determines substrate specificity. The prediction of peptide specificity is therefore the basis for most of the available computational methods aimed at predicting substrates of protein kinases. A notable exception, NetworKIN [9], uses both peptide specificity and contextual information to predict phosphorylation networks. Other currently-available prediction tools include KinasePhos [10], GPS [11], DISPHOS [12], pkaPS [13], PredPhospho [14], Scansite [15], PPSP [16] and NetPhos [17] (reviewed in [8]). These tools mine data from phosphorylation site databases, principally the phospho.ELM database [18] and employ methods that include profile hidden Markov models (KinasePhos), neural networks (NetPhos) and support vector machines (PredPhospho) to identify potential phosphorylation sites according to protein kinase family. The availability of data that links protein kinases with their substrates is a limiting factor in developing tools for substrate prediction. Furthermore, different tools use different names for protein kinase families. The lack of recognised standards for identifying protein kinases or describing their substrate interactions is an obstacle to mining data from disparate sources.

We have described a method, named Predikin, to predict protein kinase peptide specificity [19]. Predikin identifies the key residues in the protein kinase catalytic domain, termed substrate-determining residues (SDRs), which determine the sequence of the substrate heptapeptide. This method can be applied to any protein kinase sequence for which SDRs can be identified. We have successfully used Predikin to provide insight into signal transduction pathways [19,20]. It has also been used to predict phosphorylation sites, of which a number have been confirmed experimentally, in a range of biological systems [21-30]. Predikin was originally written in Javascript and made available as a web application with limited functionality. Here, we describe a new implementation of Predikin with enhanced features for the analysis of protein kinases and their substrates. We also introduce PredikinDB, an database of phosphorylation sites derived by semi-automated mining of UniProt. PredikinDB is a useful standalone resource and is also used in Predikin to generate specificity rules and prediction scores.

## Implementation

Predikin consists of two components that work together: a database of phosphorylation sites, in which the sequences of protein kinase catalytic domains and their substrates are linked and a Perl module, which queries the database to generate substrate predictions based on the features of a query kinase.

### The PredikinDB database

**Figure 1:**
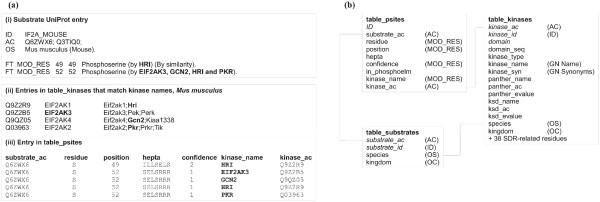
**Design and construction of the PredikinDB database**. (a) Illustration showing how a UniProt entry is parsed to link protein kinase sequences (names in bold) with phosphorylation sites. (b) PredikinDB table schema showing links between fields. Field headers in italics are primary keys. Abbreviations in parentheses indicate the UniProt line from which the field was derived. For clarity, 38 fields containing key protein kinase residues used in substrate prediction are summarised as one field.

To predict phosphorylation sites in a query kinase, a dataset of substrates and their associated kinases is required. We constructed PredikinDB, a custom database of phosphorylation sites derived from UniProt records using BioPerl-based parsers. The construction of PredikinDB is outlined in Figure 1, using a UniProt entry for a sequence from mouse as an example protein kinase substrate (Figure 1a, panel (a)).

PredikinDB was constructed using protein kinase and substrate records from the UniProt database. Protein kinase sequences were obtained in Swissprot format from the EBI SRS server using the search term "Dbxref_:IPR000719", corresponding to the InterPro signature of the protein kinase catalytic domain. Each file was parsed to extract the name, accession number, ID, gene synonyms and organism. Functions provided by the Predikin.pm Perl module (see next section) were used to extract the sequence of the catalytic domain(s), assign the kinase type, KSD family and PANTHER family and identify substrate-determining residues. These data were imported into a MySQL table.

Protein kinase substrate sequences were obtained in Swissprot format from the EBI SRS server using the search term "Keywords:phosphorylation". Each file was parsed to extract the substrate accession number, ID and organism. MOD_RES lines were parsed to extract the phosphorylated residue (phosphoserine, phosphothreonine or phosphotyrosine), its position, annotation confidence (certain, by similarity, probable or potential) and where present, names of the kinases acting at the phosphorylation site. The key feature of PredikinDB is the automated assignment of specific kinase sequences to their substrates (Figure 1a, panel (c)). This is achieved by comparing kinase names in the substrate UniProt MOD_RES line (*e.g. *"By GCN2") to kinase gene names and synonyms for kinase UniProt records from the same species (Figure 1a, panel (b)). The parsed data were then imported into two MySQL tables, one describing substrate proteins and the other describing phosphorylation sites. Figure 1b illustrates the links between tables in PredikinDB that describe kinases, substrates and phosphorylation sites. Table 1 summarises the current contents of PredikinDB and the number of phosphorylation sites that could be linked with a kinase sequence using this approach.

**Table 1:** Summary of current holdings in the PredikinDB database

**Statistic**	**Ser/Thr kinases**	**Tyr kinases**
Unique substrates	17,960	5,193
Unique substrates linked to a kinase sequence	707	459
Phosphorylation sites	55,044	8,100
Sites linked to a kinase sequence	1,448	887
Unique kinase sequences linked to a phosphorylation site	398	393

Phosphorylation sites in PredikinDB were also annotated according to whether they are present in phospho.ELM [18], a manually-curated database of experimentally-validated phosphorylation sites. This enables users to specify that only high-quality, validated sites be used in kinase substrate prediction, with the trade-off that fewer sites will be available. Of the phosphorylation sites in PredikinDB that are present in phospho.ELM, approximately 98% are annotated in UniProt as "experimental" or "by similarity", which indicates that the UniProt procedure for annotation of phosphorylation sites is reliable.

The scripts used to build PredikinDB allow it to be updated automatically; predictions made using Predikin should therefore improve incrementally over time as more phosphorylation sites and their protein kinases are annotated in UniProt. PredikinDB also provides a resource of paired kinase-substrate sequences for further investigation of protein kinase substrate specificity.

### The Predikin Perl module

The Predikin.pm Perl module was written to provide common methods for kinase sequence analysis and substrate prediction. The module makes extensive use of the BioPerl library [31]. Six methods are provided for the analysis of protein kinase sequences and their substrates: (i) classification of protein kinase type as a serine-threonine, CMGC (cyclin-dependent, MAP-, glycogen synthase kinase 3 and CK2-related kinases) or tyrosine kinase; (ii) classification into a Kinase Sequence Database (KSD; [32]) family; (iii) classification into a PANTHER database [33] family; (iv) location of substrate-determining residues in protein kinase catalytic domains; (v) extraction of putative phosphorylation sites from substrate sequences and (vi) scoring of phosphorylation sites using weight matrices. The implementation of Predikin in Perl provides three significant advantages compared with the original Predikin release: (i) a new approach to locate SDRs, (ii) methods to score phosphorylation sites and (iii) new data input options and filters; these methods are described in the following subsections.

#### Classification of kinase type and family

The catalytic domains of protein kinase sequences were classified by type, Kinase Sequence Database (KSD) family [32] and PANTHER family [33]. Protein kinase type (serine-threonine, CMGC or tyrosine kinase) was assigned by comparing query sequence with the Perl regular expressions:

Ser/Thr [LIVMFYC].{1} [HY].{1}D [LIVMFY]K.{2}N [LIVMFYCT]{3}

CMGC (YR|YK|FK) [ASPG] [PLIVS] [DER] [VIL]

Tyr [LIVMFYC] [^A] [HY].D [LIVMFY] [RSTAC] [^D].N [LIVMFYC]{3}

HMMs for KSD families were not available at the KSD website and so had to be built. Protein kinase sequences for each KSD family were retrieved in fasta format from the non-redundant protein database. The HMMER program hmmalign was used to generate alignments of the protein kinase catalytic domain using the Pkinase HMM profile from the Pfam database [34]. Each alignment was then used to build a HMM for the KSD family using hmmbuild and hmmcalibrate. The KSD family HMMs were used to search the query kinase sequence using hmmpfam, the output parsed and the best scoring KSD family assigned to the query.

PANTHER classification was performed using the pantherScore perl script (available at the PANTHER website) and the PANTHER library (version 6.1). The output of pantherScore was parsed and the best scoring PANTHER family was assigned to the query kinase sequence.

#### Identification of substrate determining residues

The identification of substrate-determining residues in the protein kinase catalytic domain by inspection of crystal structures has been described previously [19]. To locate these key residues in a query sequence, the HMMER program hmmsearch was used to align the sequence with the S_TKc HMM (SMART database accession number SM00220) [35]. The alignment was processed using the BioPerl Bio::AlignIO module to extract the position of the key motifs GXG, AMK, GEL, PEN, DFG and APE, from which the location and identity of each SDR was calculated. The use of HMM alignments locates substrate-determining residues accurately and reliably in a far wider range of protein kinase sequences than the previous approach, which used javascript string and pattern matching functions.

#### New substrate scoring methods

Previously, Predikin used a set of conditional rules of the form "if SDR = X then peptide residue = Y" to make substrate predictions. Predictions consisted of regular expressions describing possible combinations of amino acid residues in the predicted substrate peptide. These could then be used to search for sequences using tools such as ScanSite [15] or ScanProsite [36]. Amino acid frequencies at positions -3 to +3 in the substrate peptide were not calculated and scanning of user-defined substrate sequences was not straightforward.

**Figure 2:**
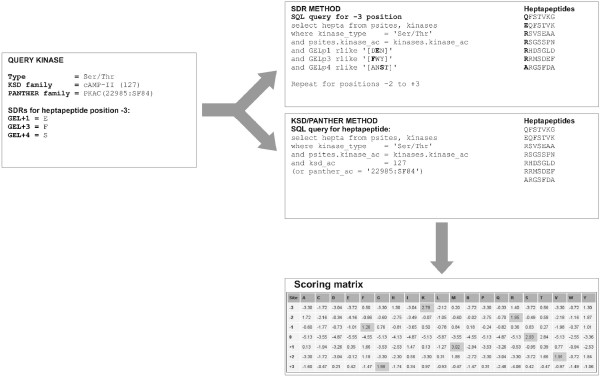
**Construction of substrate scoring matrices using SQL queries to the PredikinDB database**. Schematic showing how sequence features from a query protein kinase are used to query PredikinDB and generate Predikin scoring matrices.

**Figure 3:**
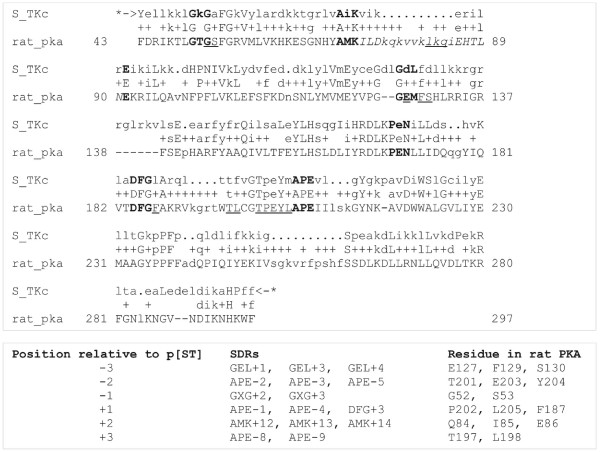
**Location of substrate-determining residues in protein kinase A using HMM alignment**. The profile HMM S_TKc from the SMART database was aligned to rat PKA (UniProt accession P27791) using the HMMER program hmmsearch. The 6 motifs used to locate SDRs are shown in bold. SDRs are underlined. The KE loop, used to determine the SDRs for the substrate +2 position is italicised. SDRs used in substrate prediction for Ser/Thr-kinases are summarised under the alignment. Position refers to the number of residues N- or C-terminal to the substrate phosphorylation site. SDRs that determine the +2 position depend on KE loop length as follows: length 12–17 = AMK+10, AMK+11, AMK+12; length 18–20 = AMK+12, AMK+13, AMK+14; length < 12 or > 20 = E-7, E-6, E-5.

Predikin now scores phosphorylation sites using matrices generated by constructing SQL queries to PredikinDB (Figure 2). Three methods of matrix generation are used. Using the SDR method, the SQL query selects substrates from PredikinDB with kinases of the same type as the query kinase, where the SDRs for positions -3 to +3 are similar to those of the query kinase (Figure 3). SDRs are considered similar if substitution using the BLOSUM62 matrix [37] gives a positive score. For example if SDR GEL+3, which determines position -3 in the substrate peptide is Ile, substrates are selected from PredikinDB where GEL+3 in the associated protein kinase sequences is any of Ile/Leu/Val/Met. The results returned by each query are used to calculate amino acid frequencies and weights (see below). As the SDR method assumes that the SDRs and each of their associated -3 to +3 positions are independent, each row of the SDR scoring matrix is calculated independently; *i.e. N*, the number of sequences used, differs for each row.

Using the KSD or PANTHER scoring methods, the SQL query selects substrates with kinases of the same type as the query kinase and of the same KSD/PANTHER family. Aligned substrate heptapeptide sequences is then used to calculate the amino acid frequency matrix at positions -3 to +3.

Frequency matrices are converted to position weight matrices using the equation:

(1) $$w(a,i)=log_{2}\frac{p(a,i)}{p(a)}$$

The background frequency of residue *a, p(a)*, is estimated as its frequency in all PredikinDB substrate sequences for each kinase type (serine-threonine, CMGC or tyrosine kinase). The frequency of a residue at position i in the substrate, *p(a,i)*, is estimated using pseudocounts by adding $\sqrt{N}$/20 to the raw frequency *f(a,i) *and dividing by *N *+ $\sqrt{N}$ (*N *= number of sequences used to calculate the frequency). This correction is not performed using the SDR score method if *N *= 0.

Predikin predictions are based on the observation that protein kinases with similar catalytic domains, in terms of either specific substrate-determining residues (SDR method) or overall similarity (KSD/PANTHER family) exhibit similar peptide specificity. The three alternative scoring methods increase the likelihood that a reliable scoring matrix can be obtained and predictions made for the majority of query kinases.

#### Scoring of phosphorylation sites

The Predikin module scores phosphorylation sites by locating all instances of the pattern *X*_3 _[*STY*]*X*_3 _in query sequences, applying the scoring matrix defined by the user (SDR, KSD or PANTHER) and converting the score to a relative value between 0–100. Potential phosphorylation sites can also be extracted from a file of input sequences (*e.g. *proteins from a complete genome) and stored in a database table for retrieval and scoring. Two filters are available to improve Predikin predictions (Table 2). Analysis of 23,209 phosphorylation sites annotated as "experimental" in PredikinDB revealed that more than 90% of sites are predicted as "disordered" by at least one of the criteria defined by DisEMBL [38] (DSSP loop/coil, hot loop or Protein Data Bank remark465) and only 0.1% are part of a transmembrane region as predicted by TMHMM [39]. These analyses therefore provide additional information that identifies likely phosphorylation sites and increases prediction accuracy. Both filters and the option of a cutoff score are available to users at the Predikin website.

**Table 2:** DisEMBL and TMHMM predictions for phosphorylation sites in the PredikinDB database

**Residue**	**Phosphorylation sites**^1^	**Disordered (%)**^2^	**TM helix (%)**^3^
S	17,575	16,596 (94.4)	6 (0.03)
T	3,705	3,371 (91.0)	6 (0.16)
Y	1 929	1,410 (73.1)	5 (0.26)
Total	23,209	21,377 (92.1)	17 (0.07)

^1^Number of phosphoresidues annotated "experimental"

^2^Number and percentage of phosphoresidues predicted as disordered using at least one DisEMBL method

^3^Number and percentage of phosphoresidues predicted as TM helix using TMHMM

Perl scripts that use the Predikin.pm module accept several command-line options which alter scoring matrix calculation and scoring of substrate sites. The main options are: (i) – disembl; score only sites predicted to be disordered using DisEMBL, (ii) – tmhmm; ignore sites predicted as transmembrane using TMHMM, (iii) – cutoff; specify a cutoff score, (iv) – first; output scores for only the first (kinase) sequence in a fasta file; (v) – noauto; do not output autophosphorylated site scores, (vi) – nokinase; do not output scores if substrate is a kinase, (vii) – noself; do not include sites of query kinase in matrix calculation and (viii) – distinct; perform homology reduction by using only non-redundant heptapeptides in matrix calculation.

#### The Predikin web interface

A new implementation of the Predikin website is available (see Availability for URL). The website was built using the Joomla open-source content management system (CMS) [40], which allows easy implementation of features such as user registration, documentation and custom forms development [41]. As the CMS is written in PHP, a PHP Perl extension [42] was employed to allow communication between the website and functions in the Predikin.pm Perl module.

At the Predikin website, users can submit a query protein kinase sequence in fasta format. They are presented with an analysis of the kinase catalytic domain(s) and scoring matrices for each of the SDR, KSD and PANTHER methods. Putative substrate sequences can then be submitted for scoring. The results for a session are stored in temporary database tables that can be exported as tab-delimited text, allowing users to build up a dataset of many substrates for each kinase. Other features of the website include tools to explore the PredikinDB database, links to related resources, extensive documentation and discussion forums.

## Results and Discussion

### Evaluation of Predikin predictions

The performance of Predikin was evaluated using receiver operator characteristic (ROC) analysis [43], implemented in the R [44] Epi package [45]. Phosphorylation sites in the PredikinDB database that were (i) linked to a kinase sequence and (ii) annotated as "experimental" or "by similarity" (2,064 sites) were obtained using a MySQL select query and the order of the returned rows was randomised. A cross-validation procedure was devised whereby the sites were divided successively ten times into a test set, containing 10% of the sites and a "training set" available to build scoring matrices, containing the remaining 90% of the sites. In addition, phosphorylation sites linked to a kinase sequence in the training set were not used to build matrices if the same kinase sequence was linked to sites present in the test set (by specifying the Predikin.pm – noself option).

For each kinase-substrate pair in the test set, Predikin SDR, KSD and PANTHER scores were calculated for all *X*_3 _[*STY*]*X*_3 _sites in the substrate, using homology reduction when building the scoring matrices. The sites were labelled as 1 (positive, an annotated site in the test set) or 0 (negative, an unannotated site) and duplicate sites (same kinase, heptapeptide, score and label) were removed. The procedure generated a set of ten files (from each test/training set combination), containing scored and labelled sites for each of the three scoring methods (SDR, KSD or PANTHER) and for each kinase type (serine-threonine, CMGC or tyrosine kinase). Each set of ten files was used as input to the ROC() method of the R Epi package and the mean AUC (area under curve) was calculated. The Epi package was also used to obtain the optimal cutoff score which maximized sensitivity; true positives/(true positives + false negatives) and specificity; true negatives/(true negatives + false positives) for each run, from which the mean sensitivity and specificity were calculated (Table 3).

**Table 3:** Area under ROC curve (AUC), sensitivity (Sn) and specificity (Sp) values for Predikin and five comparable methods

	**Ser/Thr**			**CMGC**			**Tyr**		
**Method**	**AUC**	**Sn %**	**Sp %**	**AUC**	**Sn %**	**Sp %**	**AUC**	**Sn %**	**Sp %**

**SDR**^1,2^	0.86 (0.04)	75.5 (9.2)	86.6 (7.2)	0.93 (0.02)	89.4 (2.9)	91.3 (2.1)	n/a	n/a	n/a
**KSD**^2^	0.86 (0.05)	73.7 (10.1)	90.0 (9.3)	0.88 (0.02)	83.8 (3.5)	94.1 (1.1)	0.76 (0.07)	73.0 (13.3)	79.7 (17.9)
**PANTHER**^2^	0.88 (0.04)	74.6 (5.0)	94.2 (1.8)	0.91 (0.03)	85.5 (6.4)	93.4 (2.1)	0.66 (0.09)	61.0 (5.5)	79.9 (13.8)
**GPS**	0.83	76.0	87.2	0.94	97.8	89.8	0.72	56.0	88.2
**KinasePhos**	0.78	52.9	92.5	0.95	90.8	86.6	0.89	80.0	85.1
**NetPhosK**	0.90	86.3	78.8	0.57	16.8	95.4	0.68	60.0	71.4
**PPSP**	0.92	92.2	83.6	0.95	97.8	89.5	0.81	60.0	98.1
**Scansite**	0.95	86.3	93.3	0.94	94.6	87.8	0.70	64.0	93.2

^1^SDR method not applicable to Tyr kinases

^2^AUC, sensitivity and specificity values are the mean and standard deviation (in parentheses) of 10 cross-validation tests

Predikin was compared to five commonly-used web-based tools that predict phosphorylation sites (NetPhosK, KinasePhos, GPS, PPSP and Scansite). First, the kinase families common to each method and the PANTHER accession for each family were identified (see additional file 1). Substrates of kinases with the appropriate PANTHER accession were obtained from the PredikinDB database. This procedure identified 40, 109 and 19 substrates of 27, 14 and 6 serine-threonine, CMGC and tyrosine kinases, respectively, for input to the five web servers.

The other tools cannot be run locally and do not offer convenient programmatic web access. Therefore, a Perl script was written using the Perl HTML::Form module, to submit substrate sequences to each web server, parse the output and obtain phosphorylation site scores for the corresponding kinase family. Where the option to set cutoff scores was available, the minimum value was chosen to return as many scored *X*_3 _[*STY*]*X*_3 _sites as possible. The output from each method was parsed to obtain sites that were scored by all five methods and the sites were labelled as 1 (known site in PredikinDB) or 0 (unknown site). Duplicate sites (same kinase, heptapeptide, score and label) were removed. The final output from the procedure was a set of five files (one for each method), for each of the three kinase types (serine-threonine, CMGC and tyrosine kinase), containing scores and labels for each *X*_3 _[*STY*]*X*_3 _site. Each file was then used as input to the ROC() function of the R Epi library. Existing methods cannot be fully evaluated (as training data are unavailable); therefore single AUC, sensitivity and specificity values are reported for these methods (Table 3).

The performance of each method varied according to kinase type, ranging from AUC values of 0.95 (Scansite, serine-threonine kinases; KinasePhos and PPSP, CMGC kinases) to 0.57 (NetPhosK, CMGC kinases). Mean AUC values for the Predikin methods span a similar range (0.66–0.93). Predikin performed particularly well in identifying known phosphorylation sites of CMGC kinase substrates. Predikin could therefore be said to be comparable or better than existing methods, depending on kinase type. However, comparison of Predikin with other methods is difficult and of limited value. This is principally because of the different methodology employed. Whereas Predikin calculates scoring models "on the fly" based on kinase *sequence*, other tools use pre-calculated models for a limited set of kinase *families*. The main strength of Predikin lies in its ability to score phosphorylation sites based on features of the query kinase sequence, without preclassification into kinase family.

AUC values for tyrosine kinase substrates were consistently lower than those for substrates of serine-threonine and CMGC kinases for all methods under comparison. The limited number of tyrosine kinase structures with a bound substrate in the PDB has so far precluded reliable identification of SDRs in tyrosine kinases. The binding mode of substrate peptides to tyrosine kinases is also known to differ somewhat from that of serine-threonine kinases [46]. Our comparative analysis suggests that the sequence and structure of the catalytic domain in tyrosine kinases is a less effective, but still useful predictor of peptide binding specificity than that of serine-threonine and CMGC kinases.

Predikin scores are therefore good discriminators of true phosphorylation sites. However, the effective use of Predikin requires some interpretation on the part of the user. In the following sections, we illustrate two common usage scenarios for Predikin.

#### Best substrate for a kinase

To predict the best substrate for a kinase, a user submits one kinase sequence and several putative substrate sequences. Predikin output sorted by score indicates which substrates are the most likely targets of the protein kinase. An example is provided by the protein kinase CLA4, a PAK/STE20 kinase from *S. cerevisiae*. 163 putative targets for CLA4 have been identified using a genetic screen (Brenda Andrews, personal communication) and we have applied Predikin to these data to predict the best substrates for CLA4. Interestingly, the site with the equal-highest Predikin score for CLA4 was Thr727 located in the activation loop of CLA4 itself (Table 4). This residue is not annotated as autophosphorylated in UniProt. However, autophosphorylation of threonine residues in the activation loop has been described for other PAK/STE20 kinases [47-49]. Our Predikin prediction and literature evidence strongly suggest that yeast CLA4 undergoes autophosphorylation.

**Table 4:** Predikin scores for two usage cases

**Substrates for *S. cerevisiae *kinase CLA4**^1^				**Kinases for *S. pombe *Rpb1 SPTSPSY**^2^	
**Substrate**	**Position**	**Heptapeptide**	**Score**	**Kinase**	**Score**

CLA4	727	KRATMVG	92.93	NP_592843	86.62
YOL113W	541	KRATMVG	92.93	NP_594393	84.73
YHL021C	129	KGSSFVS	91.87	NP_595739	81.60
YKR010C	527	KRNSITE	91.70	NP_595616	81.60
YNL049C	526	RATSFFG	90.14	NP_595629	81.60
YDL056W	477	KRKSTTP	88.70	NP_587921	81.60
YOL157C	527	KLFSFTK	88.25	NP_596349	81.60
YBR198C	157	RAYSMLK	87.71	NP_595795	68.41

^1^Top 8 Predikin scores (SDR method) from a set of 163 putative substrates for protein kinase CLA4 (UniProt accession P48562) from *S. cerevisiae*

^2^Top 8 Predikin scores (KSD method) for kinases at SPTSPSY repeats in substrate Rpb1 (UniProt accession P36594) from *S. pombe*

#### Best kinase for a substrate

The addition of a scoring scheme to Predikin allows the prediction of the best kinase for a substrate. In this case, the user submits one substrate sequence and several putative kinase sequences. Sorting the Predikin output by score and optionally by phosphorylation site position indicates the kinase most likely to act at each site in the substrate. To demonstrate this approach we examined the RNA polymerase II large subunit Rpb1 (UniProt accession number P36594) from the fission yeast *Schizosaccharomyces pombe*. The C-terminal domain of Rpb1 contains multiple tandem heptad repeats with the consensus sequence SPTSPSY and is extensively phosphorylated during transcription [50]. We extracted 99 putative protein kinases from the genome sequence of *S. pombe *and used Predikin to score potential phosphorylation sites in Rpb1 for each protein kinase. Rbp1 contained 10 pairs of repeats that exactly matched the SPTSPSY sequence. Seven protein kinases with high scores for SPTSPSY sites were identified (Table 4). The second ranked protein kinase Lsk1 (RefSeq accession number NP_594393) has recently been experimentally verified as the physiological partner of Rpb1 [51]. All of the high-scoring protein kinases are CMGC kinases of the CDK/MAPK family and could plausibly substitute for one another. Predikin also generated higher scores for heptapeptide SPTSPSY, centred on Ser4, than for heptapeptides centred on Ser1, Thr3 or Ser6 (data not shown). This is in agreement with the observation that the central Ser residue in the SPTSPSY motif phosphorylates most readily [50].

## Conclusion

The revised Predikin code contains numerous enhancements and new features compared with the original implementation. Predikin now features (i) a comprehensive, continuously-updated database linking protein kinases with phosphorylation sites; (ii) an SQL query-based system that generates amino acid frequency matrices for substrate peptides "on the fly", replacing the old heuristic Predikin rules; (iii) prediction scores based on SDRs or protein kinase family; (iv) improved prediction reliability through the use of profile HMMs to locate SDRs and filters to screen putative phosphorylation sites and (v) an improved web interface. The new features provide a range of user applications such as predicting the best substrates for a protein kinase, the best protein kinases for a substrate and the prediction of protein kinase-substrate interactions in large datasets such as genome sequences. Predikin remains, to our knowledge, the only system that predicts protein kinase peptide specificity for uncharacterised protein kinases from sequence alone.

## Availability and requirements

• Project name: Predikin

• Project home page: 

• Operating system: Platform-independent

• Programming language: Perl, PHP

• Other requirements: web browser. Instructions for standalone use available on request; the Predikin.pm module is heavily customized for local use and requires numerous accessory packages

• License: code available on request; Creative Commons 3.0 license

• Any restrictions to use by non-academics: licence required for commercial use; available at the Predikin website

## Abbreviations

SDR: substrate-determining residue; CMGC: cyclin-dependent/MAP/glycogen synthase kinase 3/CK2-related kinase; KSD: Kinase Sequence Database; PANTHER: Protein Analysis Through Evolutionary Relationships; CMS: content management system; ROC: receiver operator characteristic; AUC: area under ROC curve.

## Authors' contributions

NFWS wrote the Predikin code, analysed the data and wrote the manuscript. RIB and BEK developed the original Predikin concept. TH and BEK participated in the design and coordination of the study. All authors contributed to, read and approved the final manuscript.

## Supplementary Material

Additional file 1 (Table 5) – kinase families common to NetPhosK, KinasePhos, GPS, PPSP, Scansite and linked to known phosphorylation sites in PredikinDB. Kinase names listed are as defined by each program. Click here for file
