# Supplementary material for: Predikin and PredikinDB: a computational framework for the prediction of protein kinase peptide specificity and an associated database of phosphorylation sites
Source: BMC Bioinformatics. 2008 May 26;9:245. doi: 10.1186/1471-2105-9-245 (PMC2412879; doi:10.1186/1471-2105-9-245)
Supplement: Additional file 1 — (Table 5) – kinase families common to NetPhosK, KinasePhos, GPS, PPSP, Scansite and linked to known phosphorylation sites in PredikinDB. Kinase names listed are as defined by each program. [file 1471-2105-9-245-S1.pdf]

Additional file 1: Table 5. Kinase families common to NetPhosK, KinasePhos, GPS, PPSP, Scansite and linked to known phosphorylation sites in PredikinDB. Kinase names listed are as defined by each program.

**Type**	**PANTHER**	**PredikinDB**	**NetPhosK**	**KinasePhos**	**GPS**	**PPSP**	**Scansite**
**Ser/Thr**	11909/18	CSNK1E	CKI	CKI	CK1	CK1	Casein Kinase 1
	22982/64	CAMK2A	CaM-II	CaM-II	Cam-II	CAM-II	Calmodulin Dependent Kinase 2
	22985/69	PKB	PKB	PKB	PKB	PKB	Akt Kinase
	22985/86	PRKCA	PKC	PKC	PKC	PKC	PKC alpha/beta/gamma
**CMGC**	11295/61	MAPK	p38MAPK	MAPK	MAPK	MAPK	p38 MAPK
	11295/109	CDC2	cdc2	cdc2	P34CDC2	P34CDC2	Cdc2 Kinase
	11295/110	CDK5	cdk5	CDK	CDKs	CDKS	Cdk5 Kinase
**Tyr**	23256/255	SRC	SRC	Src	SRC	SRC	Src Kinase
	23256/286	EGFR	EGFR	EGFR	EGFR	EGFR	EGFR Kinase
